# Lightweight 3D Multi-Object Tracking via Collaborative Camera and LiDAR Sensors

**DOI:** 10.3390/s25237351

**Published:** 2025-12-03

**Authors:** Dong Feng, Hengyuan Liu, Zhiyu Liu

**Affiliations:** 1 School of Computer Science, Peking University, Beijing 100871, China; 2 School of Data Science, Qingdao University of Science and Technology, Qingdao 266061, China; liuhengyuan@mails.qust.edu.cn; 3 School of Information Science and Engineering, Ocean University of China, Qingdao 266100, China; lzy1280@ouc.edu.cn

**Keywords:** deep learning, computer vision, multi-object tracking, autonomous driving, ghost trajectory, data association, trajectory management

## Abstract

With the widespread adoption of camera and LiDAR sensors, 3D multi-object tracking (MOT) technology has been extensively applied across numerous fields such as robotics, autonomous driving, and surveillance. However, existing 3D MOT methods still face significant challenges in addressing issues such as false detections, ghost trajectories, incorrect associations, and identity switches. To address these challenges, we propose a lightweight 3D multi-object tracking framework via collaborative camera and LiDAR sensors. Firstly, we design a confidence inverse normalization guided ghost trajectories suppression module (CIGTS). This module suppresses false detections and ghost trajectories at their source using inverse normalization and a virtual trajectory survival frame strategy. Secondly, an adaptive matching space-driven lightweight association module (AMSLA) is proposed. By discarding global association strategies, this module improves association efficiency and accuracy using low-cost decision factors. Finally, a multi-factor collaborative perception-based intelligent trajectory management module (MFCTM) is constructed. This module enables accurate retention or deletion decisions for unmatched trajectories, thereby reducing computational overhead and the risk of identity mismatches. Extensive experiments on the KITTI dataset show that the proposed method outperforms state-of-the-art methods across multiple performance metrics, achieving Higher Order Tracking Accuracy (HOTA) scores of 80.13% and 53.24% for the Car and Pedestrian categories, respectively.

## 1. Introduction

Multi-object tracking is a technique used to associate dynamic objects across successive frames, and it plays a crucial role in computer vision and intelligent perception. It is essential for tasks like autonomous driving, surveillance, and robot navigation. Three-dimensional multi-object tracking requires the continuous and stable localization and association of multiple objects in three-dimensional space. The color images captured by camera sensors contain rich color and texture information, but their quality is significantly affected by lighting conditions and they inherently lack depth information. In contrast, the point cloud data acquired by 3D LiDAR sensors provides direct depth measurements and captures the geometric structure of objects, effectively overcoming the limitations of color cameras. This has prompted many researchers to combine cameras and 3D LiDAR for multi-object tracking. However, with the rapid advancement of 3D object detection technology and the growing complexity of application scenarios, existing 3D multi-object tracking methods have revealed several limitations, failing to meet the rigorous requirements for tracking accuracy, efficiency, and robustness in practical applications.

**Figure 1:**
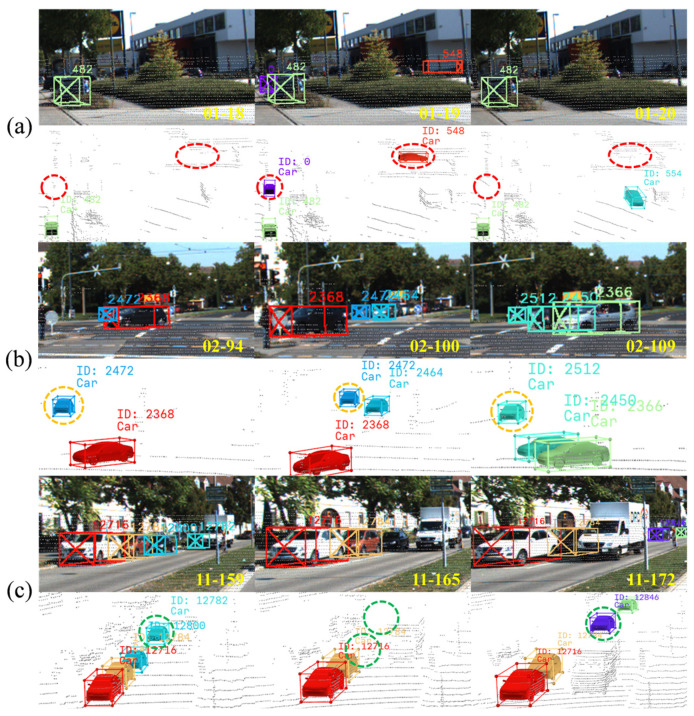
Problems in the 3D multi-object tracking task. (**a**) Consecutive false detections are incorrectly identified as real objects and assigned an ID (548). The corresponding trajectories disappear after a single frame, forming typical ghost trajectories; (**b**) The ID (2472) of a vehicle is incorrectly assigned to a neighboring vehicle during prolonged occlusion due to the baseline’s association strategy. When the original vehicle reappears, the system misidentifies it as a new object and assigns a new ID (2512); (**c**) A van passing by causes a prolonged occlusion of the vehicle with ID 12782. After the occlusion, the system fails to recognize the reappearing vehicle, mistakenly classifying it as a new object and assigning a new ID (12846).

In the 3D multi-object tracking task, existing research has generally overlooked the effective handling of false detections and ghost trajectories, which thereby affects overall tracking performance. Although some works [1,2] attempt optimization through detection result preprocessing or trajectory management strategies, they still have limitations and fail to fundamentally resolve the impact of ghost trajectories on tracking performance, as shown in Figure 1a. Furthermore, data association, a critical component of multi-object tracking, is typically performed using global matching strategies in existing methods [3,4], which calculate the matching cost between all detection results and predicted trajectories within the scene. This approach not only leads to a high redundancy in the association cost matrix and significant computational overhead, but the computational efficiency issue becomes worse, especially when multiple decision factors are introduced to improve association accuracy. Some studies [2,5] attempt to enhance matching robustness through multi-stage association strategies, but this increases both the risk of incorrect matches and the computational burden, as shown in Figure 1b. Finally, in trajectory management, existing methods [6,7] struggle to accurately determine whether an unmatched trajectory corresponds to a temporarily occluded object or one that has exited the scene. Most methods adopt a uniform strategy of keeping trajectories for a fixed number of frames, which increases both the risk of incorrect matches and computational costs. Moreover, the handling of objects with long-term occlusion is inadequate, as illustrated in Figure 1c.

To address the aforementioned issues, we propose a lightweight 3D multi-object tracking method via collaborative camera and LiDAR sensors. Our contributions are primarily as follows:

- We propose a confidence inverse normalization guided ghost trajectories suppression method. This method restores the original confidence distribution by reversing the normalization of detection result confidence. This enables effective discrimination between true objects and false detections, thereby mitigating the performance degradation caused by false detections induced by complex illumination and dynamic backgrounds. We also introduce virtual trajectories and an activation threshold strategy. Based on the confidence of virtual trajectories, we dynamically adjust their confirmation cycle, significantly reducing ghost trajectories caused by false detections while reducing the risk of trajectory loss.
- We design an adaptive matching space-driven lightweight association strategy. This strategy does not rely on computationally expensive global matching methods. Instead, it dynamically determines the matching range based on detection confidence. The detection results only build an association cost matrix with predicted trajectories within the specified range, thereby reducing computational cost and improving association accuracy. We also use Euclidean distance, relative distance, and motion direction angles to build the association cost matrix. This approach reduces computational costs while improving association accuracy, thereby meeting the real-time tracking requirements of autonomous driving and robot navigation.
- We construct a multi-factor collaborative perception-based intelligent trajectory management method. This method overcomes the limitation of retaining all unmatched trajectories. It uses factors such as trajectory position, object motion trends, and the observation distance from the data acquisition vehicle to collaboratively judge whether an unmatched trajectory corresponds to a temporarily occluded object or one that has left the scene. By extending the retention period for occluded objects and terminating trajectories of objects that have exited the scene, we effectively reduce redundant computation and mitigate identity switches caused by occlusions.

## 2. Related Works

The three main paradigms commonly used in 3D multi-object tracking are: tracking-by-detection [8,9], joint detection and tracking [10,11], and attention-based tracking [12,13]. Among these, tracking-by-detection has become the mainstream framework due to its flexibility and scalability. Its core process includes object detection, state prediction, data association, and trajectory management. Although existing tracking methods have achieved significant improvements in accuracy, they still face significant challenges when deployed in complex real-world traffic scenarios, especially in dealing with ghost trajectories, reducing data association costs, and optimizing trajectory decisions. This paper reviews the current work in three aspects: ghost trajectories, data association, and trajectory management.

### 2.1. Ghost Trajectories

Ghost trajectories are abnormal trajectories caused by false detections, typically appearing in one frame and then disappearing. They often lead to issues such as incorrect matching and identity switching. To address false detection problems, Kim et al. [14] proposed EagerMOT, which avoids false detections by projecting 3D detection results onto 2D and calculating their intersection and ratio with 2D detection results. However, this method heavily depends on the performance of the detector and tends to fail when there are discrepancies between the perception ranges of the camera and LiDAR. Pang et al. [1] proposed SimpleTrack, which uses Non-Maximum Suppression (NMS) before data association to eliminate overlapping low-quality detection results while retaining only one low-quality detection. However, this method cannot eliminate non-overlapping low-quality detections. To further enhance the robustness of NMS, Wang et al. [15] introduced YONTD-MOT, which incorporates historical trajectory confidence to guide NMS for lower-confidence prediction boxes before data association, reducing the likelihood of false detections. However, this method may mistakenly remove the true prediction box of occluded low-confidence objects. Li et al. [16] proposed Fast-Poly, which uses confidence and NMS as two independent processes to filter detection results, with accelerated computation in the NMS process. However, this method directly uses normalized confidence to filter low-quality detections, which can compress confidence score intervals, making it difficult to distinguish objects and increasing the risk of missed detections.

### 2.2. Data Association

Data association is critical for matching objects across frames and directly impacts tracking accuracy and robustness. Papais et al. [17] proposed SWTrack, which evaluates all trajectories within a sliding time window to find the most strongly correlated trajectory for association, thereby improving data association accuracy. However, this method requires iterating through all detection results and predicted trajectories in the entire scene, leading to high redundancy. To improve the discriminative power of the association matrix, Guo et al. [18] introduced a new association matrix model, which uses appearance features, object dimensions (length, width, height), and Mahalanobis distance to calculate appearance, geometry, and distance matrices, which together form the association matrix. Additionally, Zhang et al. [19] designed a more precise association matrix by enhancing the appearance matrix with additional correlated features and combining 3D GIoU (Generalized Intersection over Union) and 3D CIoU (Complete Intersection over Union) to improve the geometry matrix. However, while these methods, which incorporate multiple complex decision factors, provide more accurate information for data association and tracking, they also increase the redundancy and computational cost of the association matrix. To improve the robustness of data association, Pieroni et al. [20] proposed a three-level data association method, where each level processes detection results from different modal detectors. Zhu et al. [5] designed a four-stage data association method, where each stage determines which detection results to associate based on reliability, prioritizing the most reliable detections. While these multi-level data association methods enhance the robustness of the association strategy, they not only increase the risk of incorrect matches but also raise computational costs, impacting the real-time performance of the method.

### 2.3. Trajectory Management

Trajectory management involves post-processing the results of data association and is crucial for achieving stable tracking. Nagy et al. [21] proposed DFR-FastMOT, which addresses the long-term occlusion problem by utilizing long-term memory in the memory management module. While increasing the retention frames effectively handles long-term occlusion, it also significantly increases computational overhead. To prevent premature deletion of trajectories, which could lead to identity switching, Jin et al. [22] proposed EAFFMOT, which only deletes trajectories when there is no detection update for multiple consecutive frames. Although this method reduces identity switching to some extent, it unnecessarily retains trajectories of objects that have exited the scene, reducing tracking efficiency. To address the complexity and error-proneness of manually handling unmatched trajectories and detection methods, Sadjadpour et al. [23] proposed ShaSTA-Fuse, which extends the affinity matrix by adding four types of anchor points, thus transforming lifecycle management into an extended matching problem of the affinity matrix. While this method enhances the robustness of trajectory management, it cannot distinguish whether an object is temporarily occluded or has left the scene, leading to suboptimal performance. Doll et al. [24] proposed S.T.A.R.-Track, which introduces learnable embedding vectors to implicitly encode the trajectory lifecycle, enhancing trajectory continuity and suppressing identity switching caused by repeated initialization after brief occlusion. However, this method lacks robustness in the case of long-term occlusions or objects in complex edge regions, as its uniform retention strategy often results in the incorrect deletion of trajectories, thus triggering identity switching.

## 3. Methodology

The performance of 3D multi-object tracking in complex scenarios is often limited by several fundamental and interrelated issues. First, the confidence scores from deep learning-based detectors, after normalization, result in compressed distribution intervals, making it difficult to effectively distinguish between true objects and high-confidence false detections. These persistent false detections lead to disruptive ghost trajectories, which severely compromise tracking stability. Second, mainstream methods rely on global data association strategies, where computational costs increase quadratically with the number of objects. Moreover, introducing multiple complex decision factors further exacerbates computational redundancy, making it challenging to meet real-time application requirements. Finally, traditional trajectory management uses a “one-size-fits-all” fixed frame retention strategy, which cannot distinguish whether an object is temporarily occluded or has permanently left the scene. This leads to unnecessary computations for objects that have left the scene and identity switching caused by the premature deletion of temporarily occluded objects.

**Figure 2:**
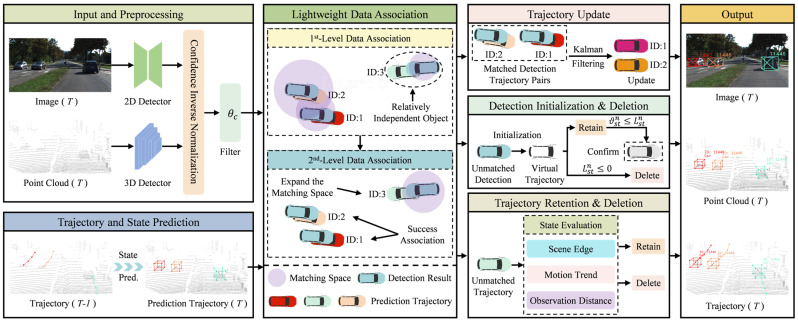
Overall framework of the proposed method.

This study proposes a lightweight 3D multi-object tracking method via collaborative camera and LiDAR sensors. The main contributions include the confidence inverse normalization guided ghost trajectories suppression (CIGTS) module, the adaptive matching space-driven lightweight association (AMSLA) module, and the multi-factor collaborative perception-based intelligent trajectory management (MFCTM) module. The overall process is shown in Figure 2. The image and point cloud data are processed by a detector to generate corresponding detection results. Then, the confidence of the detection results undergoes inverse normalization and is filtered based on a set threshold for low-confidence detections. During the data association phase, an adaptive matching space-driven lightweight association strategy is applied. The filtered detection results are matched with predicted trajectories, yielding three outputs: matched detection–trajectory pairs, unmatched detection results, and unmatched predicted trajectories. Specifically, the matched detection–trajectory pairs undergo state updates using the Kalman filter. Unmatched detection results are used to initialize virtual trajectories, which are managed according to a survival frame count strategy based on their confidence levels. The unmatched trajectories are fed into an intelligent trajectory management method with multi-factor collaborative perception for further assessment, which determines the current state of each object corresponding to an unmatched trajectory and applies targeted processing accordingly.

### 3.1. Confidence Inverse Normalization Guided Ghost Trajectories Suppression (CIGTS)

**Figure 3:**
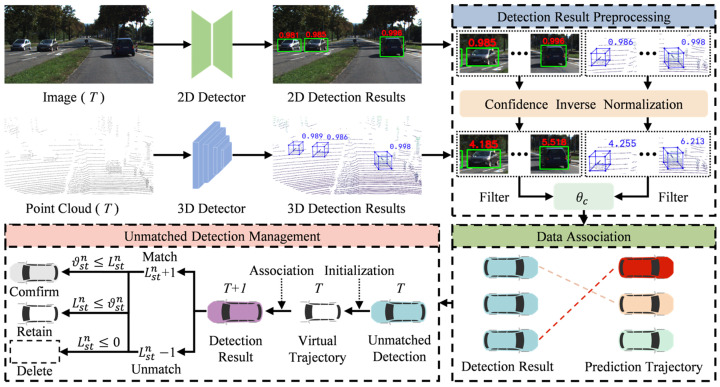
Confidence inverse-normalization-guided ghost trajectories suppression module.

Existing object detectors often generate a large number of detection results to improve recall rates, and these redundant detections can easily lead to false detections and ghost trajectories. However, most recent methods overlook the preprocessing of detection results and the suppression of ghost trajectories, resulting in suboptimal performance. To address this, as shown in Figure 3, we propose a confidence inverse normalization guided ghost trajectories suppression (CIGTS) module. This module first applies inverse normalization to the object confidence scores, restoring their original distribution to improve the distinguishability of low-quality detections. It then filters out low-quality detection results based on a preset low-confidence threshold, reducing false detections. Furthermore, leveraging the inherent difficulty for false detections to form continuous trajectories, a virtual trajectory management mechanism based on survival frame count is introduced. Specifically, for newly detected objects, a virtual trajectory with an associated survival frame count is initialized. When the survival frame count exceeds a preset activation threshold, the trajectory is converted into a formal trajectory, with different activation thresholds set based on the object’s confidence.

#### 3.1.1. Detection Result Preprocessing

Image data and point cloud data are processed separately by 2D and 3D detectors to obtain the corresponding detection result sets $D_{st}^{2d}$ and $D_{st}^{3d}$, where s denotes the current scene index and t denotes the current frame index. Since each detection result set is composed of a series of detection results, they can be further expressed as ${\left\{D_{st}^{i}\right\}}_{i=1}^{N_{s}^{t}}$ and ${\left\{D_{st}^{j}\right\}}_{j=1}^{N_{s}^{t}}$, where $N_{s}^{t}$ denotes the number of detection results in frame t of scene s. Here, $D_{st}^{i}\in R^{M\times 1}$ represents the i-th 2D detection result, and $D_{st}^{j}\in R^{N\times 1}$ represents the j-th 3D detection result, with M and N denoting the dimensionality of the detection results. The specific forms are given as: $D_{st}^{j}=\left[t,type,x_{lst}^{j},y_{lst}^{j},x_{rst}^{j},y_{rst}^{j},s_{st}^{j},h,w,l,x,y,z,r,a\right]$ and $D_{st}^{i}=\left[t,x_{lst}^{i},y_{lst}^{i},x_{rst}^{i},y_{rst}^{i},s_{st}^{i}\right]$. t is the current frame index. $\left(x_{lst}^{i},y_{lst}^{i}\right)$, $\left(x_{lst}^{j},y_{lst}^{j}\right)$ and $\left(x_{rst}^{i},y_{rst}^{i}\right)$, $\left(x_{rst}^{j},y_{rst}^{j}\right)$ are coordinates of the top-left and bottom-right corners of the 2D bounding box, respectively. $s_{st}^{i}$ and $s_{st}^{j}$ are normalized confidence scores of 2D/3D detections. type denotes object category. (h,w,l) are height, width, and length of the 3D bounding box. (x,y,z) denote center coordinates of the 3D bounding box. r denotes motion orientation angle of the object. a is observation angle of the object.

When outputting detection results, existing detectors typically apply the sigmoid function to normalize confidence scores into the range [0, 1]. However, the intervals in the original confidence score space are compressed by the nonlinear sigmoid transformation. Extreme values are pushed into the saturation regions near 0 or 1, shrinking their relative differences, while intermediate values become more concentrated. This increases the sensitivity of detection results to different threshold settings and makes it more difficult to determine appropriate confidence thresholds. Moreover, this is the main reason why directly using normalized confidence scores to filter out low-quality detections often leads to missed detections. Therefore, in order to more accurately preserve high-quality detection results while eliminating redundant or erroneous low-quality detections, we first apply inverse normalization to restore the original confidence values (taking the 3D detection results as an example), as shown in Equation (1):

(1) $${\hat{s}}_{st}^{j}=\sigma^{-1}(s_{st}^{j})=log(\frac{s_{st}^{j}}{1-s_{st}^{j}})$$

where $s_{st}^{j}$ denotes the normalized confidence score of the 3D detection result, $\sigma^{-1}(\cdot )$ represents the inverse sigmoid operation, and ${\hat{s}}_{st}^{j}$ is the restored original confidence score. By recovering the confidence scores, detections can be filtered more effectively. Specifically, we remove low-confidence detections by applying a preset threshold $\theta_{c}$, as defined in Equation (2):

(2) $${\hat{D}}_{st}^{3d}=\{D_{st}^{j}\in D_{st}^{3d}|{\hat{s}}_{st}^{j}\;\geq {\;\theta }_{c}\}$$

where $D_{st}^{3d}$ represents the original set of 3D detection results, and ${\hat{D}}_{st}^{3d}$ denotes the filtered candidate set after applying the confidence threshold.

#### 3.1.2. Virtual Trajectories

Although inverse normalization confidence filtering effectively reduces interference from low-quality detections, some false detections with relatively high confidence scores may still remain. These objects typically exhibit intermittent characteristics. if directly initialized as formal trajectories, they are prone to producing ghost trajectories. To address this issue, we introduce a virtual trajectory mechanism.

Virtual trajectories serve as candidate trajectories for formal ones. By validating the persistence and reliability of a newly detected object over a certain period, only those virtual trajectories that are continuously associated with detections are ultimately confirmed as formal trajectories. The representation of a virtual trajectory is defined as: ${\ddot{T}}_{st}^{n}=\left[t,id,type,x_{lst}^{n},y_{lst}^{n},x_{rst}^{n},y_{rst}^{n},h,w,l,x,y,z,r,{\hat{s}}_{st}^{n},L_{st}^{n}\right]$. Similar to 3D detection results, the representation includes the inverse normalization confidence score ${\hat{s}}_{st}^{n}$, while two additional state variables are introduced: id as trajectory identifier and $L_{st}^{n}$ as survival frame count of the n-th virtual trajectory in frame t of scene s. Each virtual trajectory ${\ddot{T}}_{st}^{n}$ is initialized with a survival frame count of $L_{st}^{n}$ = 1. For each subsequent frame, if the virtual trajectory ${\ddot{T}}_{st}^{n}$ is successfully matched with a detection, its survival frame count is incremented. When $L_{st}^{n}$ exceeds a predefined activation threshold, the virtual trajectory is promoted to a formal trajectory. Conversely, if a virtual trajectory ${\ddot{T}}_{st}^{n}$ fails to match with any detection, its survival frame count is decremented; once $L_{st}^{n}$ = 0, the virtual trajectory is deleted.

In real-world scenarios, low-confidence objects are more likely to be false detections compared to high confidence objects. Therefore, it is unreasonable to assign a uniform activation threshold ϑ to all virtual trajectories. To this end, we propose a confidence-aware stratified activation strategy, which suppresses ghost trajectories while reducing trajectory loss. Specifically, as shown in Equation (3), virtual trajectories are divided according to a confidence stratified threshold $\theta_{l}$. For virtual trajectories with confidence scores greater than or equal to $\theta_{l}$, the activation threshold is set to δ; otherwise, it is set to ξ:

(3) $$\vartheta_{st}^{n}=\left\{\begin{array}{c} \begin{array}{cc} \delta , & {\hat{s}}_{st}^{n}\geq \theta_{l} \end{array}\\ \begin{array}{cc} \xi , & {\hat{s}}_{st}^{n}<\theta_{l} \end{array} \end{array}\right.\;where\;\delta >\xi$$

where $\vartheta_{st}^{n}$ denotes the activation threshold of a virtual trajectory. This differentiated activation strategy can effectively suppress ghost trajectories while ensuring the completeness of true object trajectories as much as possible.

### 3.2. Adaptive Matching Space-Driven Lightweight Association (AMSLA)

**Figure 4:**
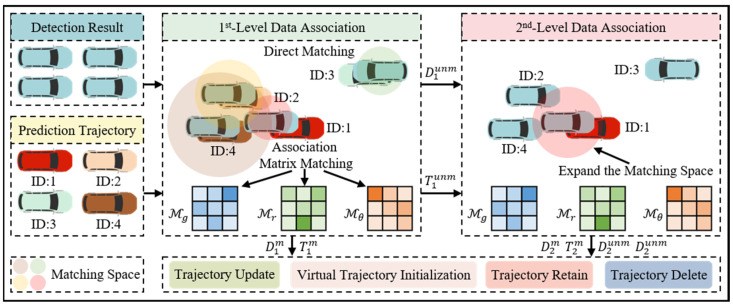
Adaptive matching space-driven lightweight data association module.

Data association is the core task in multi-object tracking. Existing methods enhance the accuracy of data association through global association, the introduction of multiple complex decision factors, and the design of multi-stage association strategies, but they tend to have poor real-time performance due to their high computational costs. To address this, as shown in Figure 4, we propose an adaptive matching space-driven lightweight association (AMSLA) module. In this module, detection results are only associated with predicted trajectories that fall within the same matching space, thus avoiding the high computational costs of global association. Additionally, this module does not rely on unstable factors such as the aspect ratio of bounding boxes or appearance information with complex feature engineering for constructing the association matrix. Instead, it utilizes the intrinsic data characteristics of the object, using the motion direction angle and relative distance (the absolute difference in distance between the object and the data acquisition vehicle) as decision factors. This allows for reducing computational costs while maintaining accuracy.

The data association task involves matching detection results and predicted trajectories, followed by dividing the matched results into corresponding matching spaces based on the object’s trajectory ID. The specific form of the predicted trajectory is as follows: ${\hat{T}}_{st}^{n}=\left[t,id,type,{a,x}_{lst}^{n},y_{lst}^{n},x_{rst}^{n},y_{rst}^{n},h,w,l,x,y,z,r,{\hat{s}}_{st}^{n}\right]$. a represents the observation angle. The state representation of a virtual trajectory is consistent with that of the detection results. After obtaining the detection results and predicted trajectories, a matching space is first determined for each detection result, whose matching radius is denoted as R, and then the association matching is performed with the predicted trajectory in that space. Since high-confidence objects have relatively stable motion states, while low-confidence objects may exhibit issues such as drift, the matching space for high-confidence detection results is set smaller, while the matching space for low-confidence detection results is set larger, as shown in Equation (4):

(4) $$R_{st}^{n}=R_{min}+(\frac{s_{st}^{max}-{\hat{s}}_{st}^{j}}{s_{st}^{max}-s_{st}^{min}})\cdot (R_{max}-R_{min})$$

where $R_{st}^{n}$ and ${\hat{s}}_{st}^{j}$ represent the corresponding matching radius and confidence of detection results. $R_{min}$ and $R_{max}$ represent the minimum and maximum matching radii, based on the statistical results of the object’s relative movement. We set $R_{min}$ and $R_{max}$ to 3 and 3.5, respectively. $s_{st}^{max}$ and $s_{st}^{min}$ represent the maximum and minimum confidence scores in the scene s with the t-th frame.

After determining the matching space, to establish a connection between the detection results and the predicted trajectories in the surrounding environment, we use the Euclidean distance to construct a global distance matrix. The center coordinates of the object are denoted as (x,y,z), as shown in Equation (5):

(5) $$M_{g}(m,n)=\sqrt{{\left(x_{m}-x_{n}\right)}^{2}+{\left(y_{m}-y_{n}\right)}^{2}+{\left(z_{m}-z_{n}\right)}^{2}}$$

where $M_{g}(m,n)$ represents the global distance matrix, ($x_{m},y_{m},z_{m}$) and ($x_{n},y_{n},z_{n}$) represent the center point coordinates of two different detection results (or predicted trajectories) in the current frame. Then, the detection results and predicted trajectories in each matching space can be filtered according to the matching radius and the global distance matrix.

During data association, if a matching space contains only one detection and one trajectory, this indicates that the object is well-separated from all other objects in the scene. A direct match can be applied without further calculation. Conversely, if the matching space contains multiple detections or predicted trajectories, it is then necessary to construct an association cost matrix for all detections and trajectories within that space.

We enhance the accuracy of data association by improving the discriminative power of the association matrix. Specifically, we use the previously constructed global distance matrix as the association matrix. Furthermore, we observe that the relative distance between an object and the data acquisition vehicle can effectively distinguish different objects, even when occlusion or deformation occurs. Therefore, we also incorporate this computationally efficient relative distance to construct the association matrix, where the distance between the object and the data acquisition vehicle is denoted as z, as shown in Equation (6):

(6) $$M_{r}(m,n)=|z_{m}-z_{n}|$$

where $M_{r}(m,n)$ represents the relative distance matrix, $z_{m}$ and $z_{n}$ represent the distances from two distinct detection results (or predicted trajectories) in the current frame to the data acquisition vehicle, respectively. However, in real-world scenarios, roads are often multi-lane and support bidirectional traffic. Relying solely on relative distance fails to distinguish between objects that are close in proximity but moving in different directions. Therefore, to further enhance the discriminative power of the association matrix, we introduce the motion direction angle r from the object (or trajectory) state information to construct the association matrix, as shown in Equation (7):

(7) $$M_{\theta }(m,n)=min(\left|r_{m}-r_{n}\right|,\;2\pi -\left|r_{m}-r_{n}\right|)$$

where $M_{\theta }(m,n)$ represents the motion direction matrix, and $r_{m}$ and $r_{n}$ represent the motion direction angles of the two different detection results (or predicted trajectories) in the current frame. As mentioned earlier, when the detection results or predicted trajectories in the matching space are not unique, we will consider the overall Euclidean distance, relative distance, and motion direction angle for the association. The weight coefficients corresponding to the global distance matrix, relative distance matrix, and motion direction matrix are denoted as α, β, and γ, respectively, as shown in Equation (8):

(8) $$M\left(m,n\right)={\alpha \cdot M}_{g}\left(m,n\right)+\beta \cdot M_{r}\left(m,n\right)+{\gamma \cdot M}_{\theta }(m,n)$$

where $M\left(m,n\right)$ denotes the weighted comprehensive association cost matrix. The weight factors satisfy 0 < α,β,γ < 1 and α + β + γ = 1. Based on experimental data, the weight factors α,β,γ are set as 0.4, 0.3 and 0.3, respectively.

Using the comprehensive association cost matrix, data association is performed within the matching space. Successfully matched detection–trajectory pairs are removed from all matching spaces, with their corresponding matching spaces being deleted accordingly. Upon completing the data association process, four distinct outcomes are identified: matched detections $D_{1}^{m}$, matched trajectories $T_{1}^{m}$, unmatched detections $D_{1}^{unm}$, and unmatched trajectories $T_{1}^{unm}$. Considering that real-world scenarios may involve objects with significant inter-frame displacement, we proceed to conduct a second stage of data association specifically for the unmatched detections and trajectories. Specifically, we expand the matching space for unmatched detections to μ times its original size, while also incorporating 2D detection results to address unmatched trajectories caused by omissions from the 3D detector. After the secondary data association, we similarly obtain four outcomes: matched detections $D_{2}^{m}$, matched trajectories $T_{2}^{m}$, unmatched detections $D_{2}^{unm}$, and unmatched trajectories $T_{2}^{unm}$. Finally, the matched detections ($D_{1}^{m}$,$D_{2}^{m}$) and matched trajectories ($T_{1}^{m}$,$T_{2}^{m}$) from both the first and second data association stages, along with the unmatched detections $D_{2}^{unm}$ and unmatched trajectories $T_{2}^{unm}$ from the second association stage, are fed into the trajectory management module for operations such as updating, initializing, and terminating trajectories.

### 3.3. Multi-Factor Collaborative Perception-Based Intelligent Trajectory Management (MFCTM)

**Figure 5:**
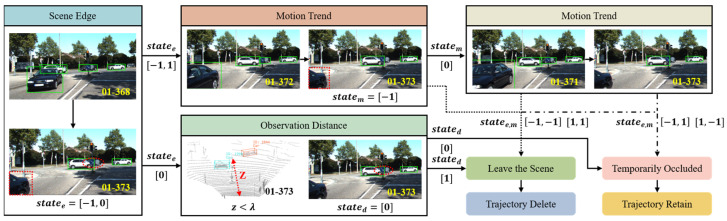
Multi-factor collaborative perception-based intelligent trajectory management module.

Trajectory management aims to process the multiple outcomes of data association, preventing identity switches and trajectory fragmentation. However, existing methods fail to distinguish between occluded objects and objects that have left the scene. They uniformly retain unmatched trajectories for a fixed number of frames, which not only increases computational overhead but also leads to issues such as erroneous matches. To address this, as shown in Figure 5, we propose a multi-factor collaborative perception-based intelligent trajectory management (MFCTM) module. This module first uses the 2D bounding box coordinates of unmatched trajectories to determine whether the corresponding object is at the boundary of the scene. Then, it combines the motion trend analysis of two consecutive frames’ bounding boxes to identify the direction of the object’s movement, further distinguishing whether the object at the scene’s boundary is entering or leaving the scene. Finally, for objects far from the scene’s boundary, it uses the observation distance between the data acquisition vehicle and the object (the depth information z from the 3D bounding box center coordinates) to determine if the object has moved beyond the perception range and has left the scene. Through this multi-factor collaborative perception mechanism, the trajectory of objects that have left the scene can be promptly terminated to reduce computational load, while reliably retaining the trajectories of occluded objects to wait for their reappearance. This approach effectively reduces the number of identity switches and improves overall tracking efficiency.

#### 3.3.1. Trajectory Management

The adaptive matching space-driven lightweight association module outputs six sets of results to the trajectory management module. For the matched detection–trajectory pairs $D_{1}^{m}\_T_{1}^{m}$ and $D_{2}^{m}\_T_{2}^{m}$, Kalman filtering is applied to update the corresponding trajectories $T_{1}^{m}$ and $T_{2}^{m}$ using the detection results $D_{1}^{m}$ and $D_{2}^{m}$, respectively. To account for false detections in the detection results, a virtual trajectory is initialized for the unmatched detection result $D_{2}^{unm}$. It is converted into a formal trajectory only when its survival frame count exceeds the activation threshold. For the unmatched trajectories $T_{2}^{unm}$, the object state determination strategy is employed to determine whether the corresponding object is temporarily occluded or has left the scene. Since trajectories represent the historical output of detection results, the object’s status in the current frame can be determined based on the trajectories. The decision process is as follows: (1) determine whether the trajectory is located at the scene boundary; (2) determine the motion trend of the trajectory; (3) determine whether the trajectory exceeds the perception range. If the object corresponding to the unmatched trajectories $T_{2}^{unm}$ has left the scene, it is immediately deleted to reduce the computational overhead of retaining trajectories. Otherwise, the object is considered occluded, and a larger survival frame count φ is set for it. When the number of consecutive unmatched frames exceeds φ, the corresponding trajectory is deleted. This enhances the ability to handle long-term occlusions and reduces identity switches.

#### 3.3.2. Object State Determination

**Scene Boundary:** We observe that all objects consistently exit the scene from either the left or right side. Therefore, whether an unmatched trajectory corresponds to an object that has left the scene can be determined by assessing its position relative to the scene boundary. The 2D bounding box coordinates within a trajectory contain spatial information about the object’s position in the scene. When the x-coordinate of the top-left corner $x_{lst}$ of the 2D bounding box corresponding to an unmatched trajectory is approximately 0, or the x-coordinate of the bottom-right corner $x_{rst}$ is approximately $w_{max}$ (the maximum scene width), it indicates that the object is located at the scene boundary or has partially exited the scene. In such cases, the most likely reason for the trajectory being unmatched is that the corresponding object has already left the scene. We introduce a small value ϵ (3 pixels) to avoid noise interference. The specific decision process is shown in Equation (9):

(9) $${state}_{e}=\left\{\begin{array}{cc} -1, & if\;x_{lst}-0\leq \epsilon \\ 1, & if\;|x_{rst}-w_{max}|\leq \epsilon \\ 0, & else \end{array}\right.$$

where ${state}_{e}$ represents the boundary state of the trajectory. −1 indicates that the trajectory is located at the left boundary, 1 indicates the right boundary, and 0 signifies the trajectory is far from any boundary. For unmatched trajectories at the scene boundary, they are not immediately deleted; instead, their motion trend is further analyzed. Similarly, trajectories far from the boundary are not immediately retained; rather, their observation distance is further evaluated.

**Motion Trend:** In real-world scenarios, there are cases where an object is quickly occluded after entering the scene. If the trajectory is judged solely based on whether it is located at the scene boundary, the corresponding trajectory of the object will be deleted, and when the occlusion ends and the object reappears in the scene, this will lead to an identity switch. Therefore, we introduce motion trend to further determine whether the object is entering or leaving the scene. Specifically, when the unmatched trajectory is located at the left boundary, its top-left corner’s horizontal coordinate is fixed at 0, so we determine its motion trend based on the change in the horizontal coordinate of the bottom-right corner. Similarly, for unmatched trajectories located at the right boundary, their motion trend is determined based on the change in the horizontal coordinate of the top-left corner. We introduce a small value δ (2 pixels) to filter out interference caused by minor bounding box jitter, as shown in Equations (10) and (11):

(10) $$∆x=\left\{\begin{array}{cc} x_{rst}-x_{rst-1}, & if\;{state}_{e}=-1\\ x_{lst}-x_{lst-1}, & if\;{state}_{e}=1 \end{array}\right.$$

(11) $${state}_{m}=\left\{\begin{array}{cc} -1, & if\;∆x<-\delta \\ 1, & if\;∆x>\delta \\ 0, & else \end{array}\right.$$

where $x_{rst}$, $x_{lst}$ and $x_{rst-1}$, $x_{lst-1}$ denote the x-coordinates of the bottom-right and top-left corners of the unmatched trajectory at frame t and frame t − 1, respectively; ∆x represents the offset of the trajectory between two consecutive frames; ${state}_{m}$ indicates the motion state of the trajectory: −1 denotes a leftward offset, 1 denotes a rightward offset, and 0 signifies that the motion trend is undefined and requires further judgment based on historical data, as shown in Equation (12):

(12) $$∆x=\left\{\begin{array}{cc} x_{rst}-x_{rst-2}, & if\;{state}_{e}=-1\\ x_{lst}-x_{lst-2}, & if\;{state}_{e}=1 \end{array}\right.$$

where $x_{rst-2}$ and $x_{lst-2}$ denote the x-coordinates of the bottom-right and top-left corners of the unmatched trajectory at frame t − 2, respectively. When the combined state [${state}_{e},{state}_{m}]$ is [−1, −1] or [1, 1], it indicates that the object corresponding to the unmatched trajectory is in the process of leaving the scene, and the respective unmatched trajectory is immediately deleted. Conversely, a combined state of [−1, 1] or [1, −1] indicates that the object is entering the scene, and the respective unmatched trajectory is retained to ensure it can be reassociated when the object reappears.

**Observation Distance:** In real-world scenarios, there exists a special category of objects that, although far from the scene boundary, can no longer be perceived or tracked due to the limited perception range of the detector and the excessive distance between the object and the data acquisition vehicle. Since the distance between such objects and the acquisition vehicle continues to increase in subsequent frames, they should also be considered to have left the scene, and their corresponding trajectories should be deleted. Specifically, by comparing the observation distance z of an unmatched trajectory with the perception threshold λ, it is possible to determine whether the corresponding object has moved beyond the perceivable range due to excessive distance in the current frame, as shown in Equation (13):

(13) $${state}_{d}=\left\{\begin{array}{cc} 1, & if\;z>\lambda \\ 0, & else \end{array}\right.$$

where ${state}_{d}$ denotes the distance state of a trajectory: 1 indicates that the object corresponding to the unmatched trajectory has exceeded the perception range, is considered to have left the scene, and the unmatched trajectory is deleted; 0 indicates that the object lies within the perception range, is considered temporarily occluded, and the unmatched trajectory is retained.

In summary, the proposed object state determination strategy employs a collaborative decision-making mechanism that integrates scene boundary awareness, motion trend modeling, and observation distance analysis. This multi-faceted approach accurately distinguishes between the exiting and occluded states of unmatched trajectories. Consequently, it significantly reduces computational overhead caused by redundant trajectory retention while effectively preventing identity switches resulting from premature deletion of the occluded trajectories, thereby achieving intelligent and efficient trajectory management in complex scenarios.

## 4. Experiments

### 4.1. Experiment Setting

#### 4.1.1. Datasets

To evaluate the performance of the proposed 3D multi-object tracking algorithm, we conduct systematic experimental validations on the KITTI [25] tracking benchmark, which is widely adopted in the field of autonomous driving. Recognized as one of the most authoritative evaluation platforms for 3D multi-object tracking tasks, the KITTI dataset is distinguished by its high data quality, realistic scenarios, and fine-grained annotations. For the multi-object tracking task, the KITTI benchmark provides 21 fully annotated training sequences and 29 unannotated test sequences. All sequences are captured from real-road environments, covering diverse typical driving scenarios such as urban streets, rural roads, and highways. These sequences incorporate multi-dimensional challenges including variations in object density, occlusion levels, and illumination conditions, thereby enabling a comprehensive assessment of the algorithm’s robustness and generalization capability under complex traffic conditions.

#### 4.1.2. Evaluation Metrics

We adopt a recognized and up-to-date comprehensive metric system to evaluate the tracking algorithm’s accuracy and robustness from multiple dimensions. Specifically, we employ Higher Order Tracking Accuracy (HOTA) [26] as the primary evaluation metric, as it provides a more balanced and accurate characterization of performance in complex scenarios. Furthermore, we introduce a series of metrics from the CLEAR [27] evaluation protocol for supplementary validation of the method’s effectiveness. These include: Multi-Object Tracking Accuracy (MOTA), Multi-Object Tracking Precision (MOTP), False Positives (FP), False Negatives (FN), Identity Switches (IDSW), Association Accuracy (AssA), and Localization Accuracy (LocA).

#### 4.1.3. Implementation Details

The model is implemented based on the PyTorch 1.9.1 deep learning framework utilizing Tesla V100 SXM2 GPUs from NVIDIA Corporation (Santa Clara, CA, USA). The Python and CUDA versions are 3.8.16 and 11.1, respectively. The input data consists of three parts: images, point clouds, and trajectories from the previous timestamp. The image and point cloud data are in one-to-one correspondence, comprising 21 training sequences and 29 test sequences. Specifically, the training set contains 8008 frames, while the test set contains 11,095 frames. The trajectory data are updated using a Kalman filter. The cost matrix weight coefficients α, β, and γ are set to 0.4, 0.3, and 0.3, respectively. The matching radii, $R_{min}$ and $R_{max}$, are set to 3 and 3.5, respectively. The optimal values for the confidence filtering threshold $\theta_{c}$, confidence stratified threshold $\theta_{l}$, activation thresholds δ and ξ, matching space expansion factor μ, trajectory retention frame count φ, and perception threshold λ were determined through ablation studies. The Adaptive Moment Estimation (Adam) optimizer was employed, with the initial learning rate, weight decay coefficient, and momentum set to 0.01, 0.01, and 0.9, respectively. For the 3D and 2D detectors, PointRCNN [28] and RRC [29] were used, respectively, aligning with the baseline method to ensure a fair comparison.

### 4.2. Quantitative Results

We conduct a systematic evaluation of the widely-used KITTI tracking benchmark to comprehensively validate the effectiveness of the proposed lightweight tracking method via collaborative camera and LiDAR sensors for the 3D multi-object tracking task. Given that existing studies are typically optimized for specific object categories (e.g., cars or pedestrians), we accordingly performed detailed comparisons between our method and current state-of-the-art approaches separately for both the Car and Pedestrian categories. The results demonstrate that our method outperforms existing techniques across multiple key metrics. The detailed evaluation results are presented in Table 1 and Table 2. For clarity in presenting the performance ranking, the top first, second, and third methods for each metric are highlighted in red, blue, and green, respectively. An upward arrow (↑) denotes higher values are better, while a downward arrow (↓) signifies lower values are preferable.

As evidenced by the comprehensive comparison in Table 1 and Table 2, the proposed lightweight 3D multi-object tracking method via collaborative camera and LiDAR sensors achieves highly competitive results on the KITTI test set. It demonstrates significant advantages across multiple key metrics, particularly in HOTA, AssA, LocA, MOTP, and IDSW. This superior performance is primarily attributed to the synergistic effect of the three proposed core modules. Firstly, the Confidence Inverse-Normalization-Guided Ghost Trajectories Suppression (CIGTS) module significantly enhances the discriminative capability for objects by recovering the original confidence distribution, effectively suppressing false positives and reducing missed detections. Consequently, this leads to a reduction in FP and FN metrics and an improvement in LocA. Compared to the baseline model, our method reduces the FP and FN metrics by 21.3% and 20.1%, respectively. Secondly, the Adaptive Matching Space-Driven Lightweight Association (AMSLA) module discards the redundant global matching strategy. It adaptively defines the matching range based on object confidence and constructs a highly discriminative association cost matrix by incorporating low-complexity motion and geometric features. This approach significantly improves association accuracy while substantially reducing computational costs, leading to notable improvements in the AssA and IDSW metrics. Finally, the Multi-Factor Collaborative Perception-based Intelligent Trajectory Management (MFCTM) module accurately distinguishes between occluded objects and exiting objects through multi-dimensional state assessment of unmatched trajectories. This strategy not only avoids premature deletion of valid trajectories but also promptly terminates redundant ones, thereby preventing incorrect associations. Consequently, it further reduces IDSW while simultaneously improving the MOTP metric.

The pedestrian category is characterized by smaller object size, weaker appearance features, frequent occlusion, and non-rigid motion, presenting significant challenges in distinguishing false positives from true objects, and exiting objects from temporarily occluded ones. As shown in Table 2, for the pedestrian tracking task, the proposed method achieves first place in HOTA, AssA, and IDSW metrics, and secures second place in LocA, MOTA, and MOTP metrics. These results convincingly demonstrate the effectiveness of our method in tracking non-rigid, small-scale, and easily occluded objects.

### 4.3. Ablation Experiments

To systematically evaluate the effectiveness of the proposed method, we conduct component-wise ablation studies on the KITTI training set for the three core modules: the CIGTS module, the AMSLA module, and the MFCTM module. These studies aim to quantify the contribution of each module to the overall tracking performance. The experiments employ the official KITTI benchmark evaluation metrics, including HOTA, MOTA, MOTP, FP, FN, and IDSW. Among these, HOTA, MOTA, and MOTP are positive metrics where higher values indicate better performance, whereas FP, FN, and IDSW are negative metrics for which lower values signify superior tracking results. The detailed ablation study results are presented in Table 3.

As evidenced in Table 3, incorporating the CIGTS module into the baseline model yields marked improvements across all evaluation metrics. This enhancement is primarily attributed to the module’s ability to perform inverse normalization on detection confidence, which sharpens the distinction between true objects and false positives. Concurrently, the introduction of virtual trajectories and a hierarchical activation mechanism effectively suppresses ghost trajectories caused by false positives while mitigating the risk of losing true objects due to threshold sensitivity. Replacing the original association strategy in the baseline model with the AMSLA module further elevates multiple tracking performance metrics. This improvement stems from the module’s abandonment of the computationally redundant global matching strategy in favor of adaptively defining the matching space based on detection confidence. Furthermore, it constructs a highly discriminative association cost matrix by integrating low-complexity features including Euclidean distance, relative distance, and motion direction angle, thereby significantly enhancing association accuracy while substantially reducing computational overhead. Finally, the integration of all three proposed modules into the baseline model yields the optimal overall performance. This confirms that the MFCTM module also contributes significantly to the overall tracking performance. By comprehensively leveraging multi-source information from unmatched trajectories—including 2D bounding box positions, motion trends, and observation distances—this module intelligently discriminates between temporarily occluded objects and those that have exited the scene. This capability enables precise decisions regarding trajectory retention versus termination, thereby enhancing the algorithm’s tracking robustness and efficiency in complex scenarios.

According to the results in Table 3, the contribution of each module to the computational efficiency can be clearly observed. First, the introduction of the CIGTS module yields an initial improvement in Frames Per Second (FPS). This is primarily attributed to the module’s ability to filter out a large number of low-quality detections at the source, directly reducing the computational burden for subsequent data association. Furthermore, it effectively suppresses the generation of ghost trajectories, thereby avoiding unnecessary computational overhead during the association and trajectory management stages caused by these false trajectories. Second, the incorporation of the AMSLA module leads to a significant enhancement in FPS. This is mainly because it abandons the computationally redundant global association strategy, performing associations only between detections and trajectories within the same matching space, which substantially reduces the computational complexity of the association process. Additionally, this module avoids using computationally expensive similarity measures, such as appearance information, when constructing the association cost matrix, further optimizing computational efficiency. Finally, the integration of the MFCTM module achieves the highest FPS performance. This stems from its ability to promptly terminate trajectories of objects that have exited the scene. This not only reduces the memory and computational overhead associated with maintaining a large number of active trajectories but also avoids meaningless association attempts with these trajectories in subsequent frames, thereby freeing up computational resources.

During the preprocessing of detection results, the confidence filtering threshold $\theta_{c}$ plays a critical role in distinguishing false positives from true objects. To determine the optimal value for $\theta_{c}$, we conduct an ablation study, the results of which are summarized in Table 4.

As shown in Table 4, the model achieves optimal performance across all evaluation metrics when the confidence filtering threshold $\theta_{c}$ is set to 1.4. This is primarily because an excessively large threshold would incorrectly delete true objects, leading to issues such as missed detections and association failures. Conversely, an overly small threshold would retain a portion of false positives, potentially causing ghost trajectories and identity switches. Furthermore, the value of the confidence stratified threshold $\theta_{l}$ determines the activation period distribution of virtual trajectories. To enable the model to achieve its optimal state, we also conduct an ablation study on $\theta_{l}$, with the results shown in Table 5.

As indicated in Table 5, the model achieves optimal performance when the confidence stratified threshold $\theta_{l}$ is set to 3.5. To further investigate the impact of the activation thresholds on tracking performance, we conduct separate ablation studies for the activation thresholds δ and ξ. The results are presented in Table 6 and Table 7.

As shown in Table 6 and Table 7, the tracking performance achieves the optimum when the activation thresholds δ and ξ are set to 2 and 3, respectively. This is primarily because a smaller activation threshold increases the likelihood of false detections forming ghost trajectories, thereby leading to incorrect matches and identity switches. Conversely, a larger activation threshold increases the trajectory confirmation time, which subsequently results in association failures and trajectory fragmentation.

In the data association stage, we sequentially incorporate the global distance matrix $M_{g}$, relative distance matrix $M_{r}$, and motion direction matrix $M_{\theta }$ into the proposed association module to evaluate the impact of each association matrix on tracking performance. The results are summarized in Table 8.

As shown in Table 8, incorporating the relative distance matrix $M_{r}$ into the association cost matrix improves all evaluation metrics. A more substantial performance gain is observed when the motion direction matrix $M_{\theta }$ is further added. This is primarily attributed to the fact that object motion direction can enhance the discriminative power of the relative distance criterion for distinguishing different objects, thereby improving the accuracy of data association and the robustness of tracking. Furthermore, the matching space expansion factor μ in the data association module affects the performance of the secondary data association. To explore the optimal performance of our model, we conduct an ablation study on this parameter, with the results presented in Table 9.

As shown in Table 9, the model achieves optimal performance when the matching space expansion factor μ is set to 1.5. This is primarily because an appropriate expansion factor can effectively handle large inter-frame displacements while simultaneously avoiding the introduction of incorrect matches.

**Figure 6:**
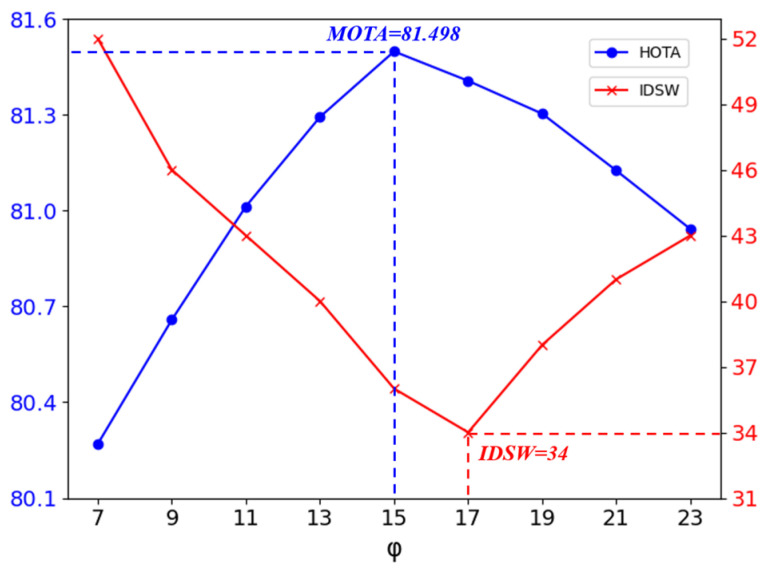
Results of the ablation study on trajectory retention frame count φ.

After the object state is determined by the multi-factor collaborative perception-based intelligent trajectory management module, unmatched trajectories corresponding to objects that have left the scene are immediately purged, while those corresponding to temporarily occluded objects are retained as much as possible to address the challenge of long-term occlusion. However, as the number of retention frames increases, the computational cost of the model rises accordingly. Furthermore, the reliability of unmatched trajectories gradually diminishes, leading to an increased probability of incorrect associations. Therefore, we conduct an ablation study on the retention frame count φ for unmatched trajectories to determine its optimal value. The results are shown in Figure 6.

As illustrated in Figure 6, the evaluation metric HOTA achieves its optimum when the retention frame count φ for unmatched trajectories is set to 15, whereas the IDSW metric reaches its minimum at φ = 17. To balance model efficiency and performance, we ultimately set the retention frame count φ to 15. Subsequently, the value of the perception threshold λ in the object state determination strategy also influences tracking performance. Therefore, we conduct an ablation study on the perception threshold λ, with the results presented in Table 10.

As shown in Table 10, the model fails to achieve optimal performance with either excessively large or small values of the perception threshold λ. This occurs primarily because an overly small λ tends to misclassify temporarily occluded objects as having left the scene, leading to the premature deletion of their unmatched trajectories. When these objects reappear, identity switches are likely to occur. Conversely, an excessively large λ tends to retain unmatched trajectories for objects that have actually left the scene, misclassifying them as temporarily occluded. This increases computational costs and elevates the risk of incorrect associations.

Most parameters in the proposed method maintain stable tracking performance when varied within a reasonable range, indicating low sensitivity to precise parameter values. Moreover, their dependencies are simple and tied to inherent physical constraints, making them insensitive to scene changes and highly generalizable across different environments.

### 4.4. Visualization Analysis

**Figure 7:**
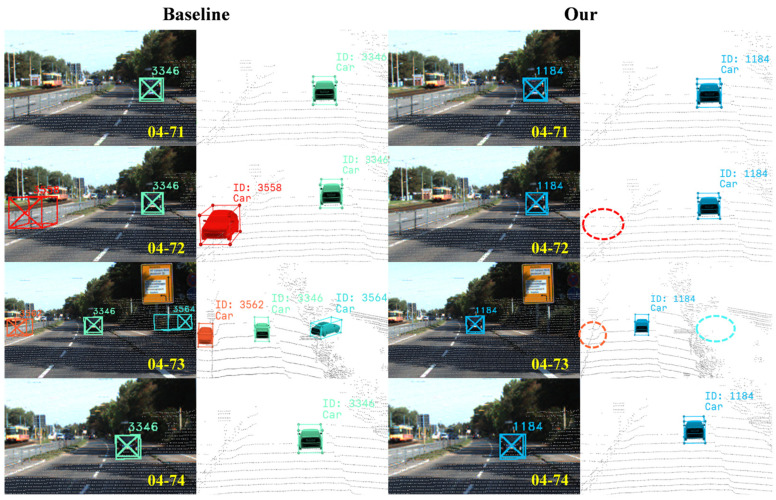
Visual comparison of ghost trajectories. The baseline model produces multiple ghost trajectories (IDs 3558, 3562, 3564) in frames 72 and 73, whereas our proposed method avoids such issues.

**Figure 8:**
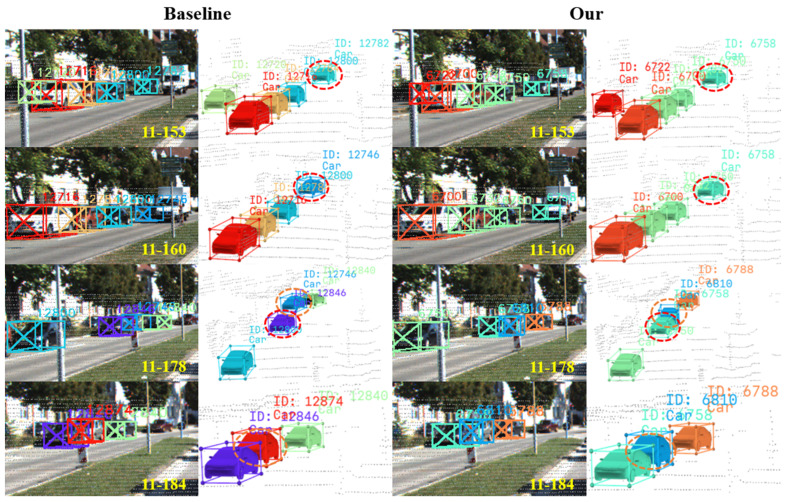
Visual comparison of data association. The cascade strategy used by the baseline model leads to ID switches during data association (e.g., ID 12782 changes to 12746 and then to 12874), while our method maintains identity consistency throughout the sequence.

**Figure 9:**
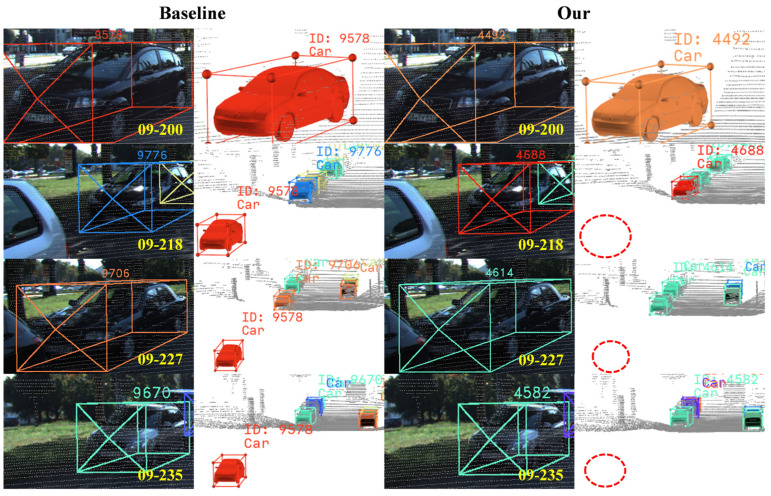
Visual comparison of trajectory retention. The baseline model incorrectly retains the trajectory of an object that has left the scene (ID 9578). Our method intelligently identifies and promptly terminates such trajectories.

To intuitively validate the effectiveness of the proposed method in addressing core challenges such as false positives, ghost trajectories, long-term occlusions, and identity switches, we conduct a systematic visual comparison of the tracking results between our method and the baseline model on the KITTI dataset. By selecting representative complex scenarios, the tracking outcomes are presented from both 2D image (left) and 3D point cloud (right) perspectives. The specific visualization results are shown in Figure 7, Figure 8 and Figure 9.

As shown in the left subfigures of Figure 7, the baseline model produces ghost trajectories in both frames 72 and 73 of the fifth scene. This occurs because it neglects the preprocessing of false positives, resulting in consecutive false detections that manifest as ghost trajectories. In contrast, the visualization results on the right demonstrate that the proposed method is completely free from ghost trajectories. This clearly indicates that our proposed CIGTS module effectively filters out false positives. Furthermore, the introduction of virtual trajectories optimizes the trajectory initialization strategy, collectively leading to effective suppression of ghost trajectories.

As shown in the left visualizations of Figure 8, the object with ID 12782 in frame 153 undergoes an identity switch in frame 160, where its ID changes to 12746. Subsequently, the object with ID 12746 in frame 160 is reassigned a new ID, 12846, by frame 178. Finally, the object identified as 12746 in frame 178 experiences another identity switch in frame 184, acquiring ID 12874. In contrast, the right visualizations demonstrate that the proposed method exhibits no identity switches. This robustness is primarily attributed to the proposed AMSLA module. This module not only reduces the risk of incorrect matching through its adaptive matching space strategy but also incorporates more stable determining factors to construct a more discriminative association matrix, thereby further enhancing the accuracy of data association.

As shown in the left visualization of Figure 9, the baseline model fails to distinguish between objects that have exited the scene and those that are temporarily occluded. It uniformly retains all unmatched trajectories for a fixed number of frames, leading to the excessive retention of an exited object (ID 9578) and causing duplicate matching in subsequent frames. In contrast, the right visualization demonstrates that the proposed method effectively avoids duplicate matching for exited objects. This capability primarily stems from the MFCTM module, which comprehensively evaluates object states through scene boundary awareness, motion trend analysis, and observation distance assessment. This integrated approach enables timely deletion of unmatched trajectories corresponding to exited objects while prioritizing the retention of those associated with temporarily occluded objects.

**Figure 10:**
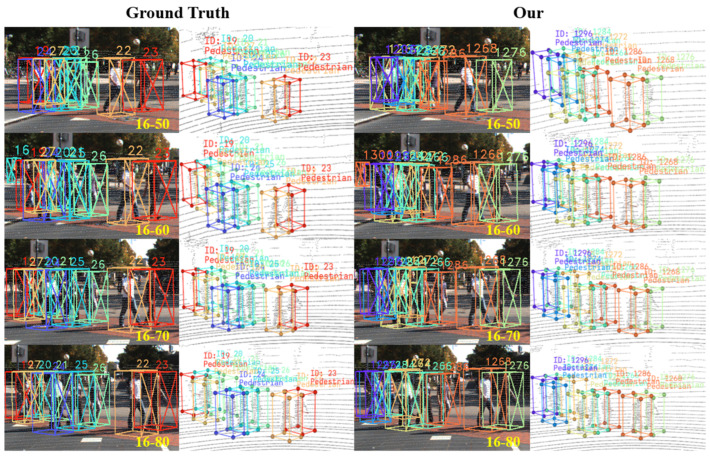
Visual comparison of pedestrian tracking results with ground truth. In dense scenarios, the comparison with ground truth across consecutive frames shows that our method does not produce any ID switches, effectively validating its tracking stability under challenging conditions.

We also conduct a visual analysis of the pedestrian tracking results on the KITTI dataset to further validate the comprehensive performance of the proposed method on the pedestrian category. We selected typical complex scenarios containing multiple objects with severe occlusion, and performed a visual comparison between the tracking results of our method and the official KITTI ground truth. This comparison objectively demonstrates the effectiveness and stability of our method in pedestrian tracking tasks. As depicted in Figure 10, the left side shows the visualization of the ground truth annotations, while the right side presents the visual output of the tracking results generated by our proposed method.

In Figure 10, we select 40 consecutive frames of tracking results from high-complexity scenes for visualization. A horizontal analysis reveals that the proposed method produces no ghost trajectories, thereby validating the effectiveness of the CIGTS module. Furthermore, a longitudinal analysis across the 40-frame sequence demonstrates the complete absence of identity switches or incorrect associations, achieving continuous and accurate tracking of multiple objects. This confirms the efficacy of the AMSLA module. From a holistic perspective, the visualization results substantiate that the proposed 3D MOT method effectively addresses critical challenges including false positives, ghost trajectories, long-term occlusions, and identity switches, thereby enabling more stable and robust object tracking.

## 5. Conclusions

This paper proposes a lightweight 3D multi-object tracking method via collaborative camera and LiDAR sensors. It aims to address challenges including false detections and subsequent ghost trajectories caused by varying illumination conditions and dynamic backgrounds, poor tracking real-time performance due to inefficient data association, and difficulties in distinguishing object states and identity switches resulting from occlusions. By introducing an inverse normalization confidence enhancement mechanism and a hierarchical virtual trajectory management strategy, our method significantly improves the ability to distinguish true objects from false detections. Furthermore, we design a lightweight association module featuring adaptive matching space and multi-discriminant factor fusion, which reduces computational overhead while enhancing matching accuracy. Additionally, a multi-factor collaborative decision-making mechanism for trajectory management is constructed to achieve precise discrimination between occluded objects and those exiting the scene. Experimental results on the KITTI dataset demonstrate that our method achieves excellent performance in tracking multiple object categories, including cars and pedestrians, and outperforms existing state-of-the-art methods, particularly on higher-order metrics such as HOTA, AssA, and IDSW.

Nevertheless, the proposed method still has certain limitations. For instance, the filtered low-quality detections might exhibit positional inaccuracies due to factors such as minor jitter. To maintain a lightweight design and align with mainstream research practices, we do not perform positional regression correction on these detections. However, in extreme cases, where a low-quality detection initially exhibits a significant positional error and remains persistently occluded in subsequent frames without updates, the accumulation of errors may eventually affect the accuracy of data association. Future work will focus on optimizing low-quality detections and enhancing association robustness. Specifically, we plan to perform position regression on low-quality detections to obtain more precise object location information, and to design an association strategy based on the possible states of objects in real-world scenarios, thereby further enhancing the robustness and practicality of the proposed method under complex environments.

## Figures and Tables

**Table 1:** Evaluation result of “Car” on KITTI test set.

Methods	HOTA↑	AssA↑	LocA↑	MOTA↑	MOTP↑	FP↓	FN↓	IDSW↓
DeepfusionMOT [2]	75.46	80.05	86.70	84.63	85.02	4601	601	84
BcMODT [19]	71.00	69.14	86.93	85.48	85.31	3353	1260	381
Seg2Track-SAM2 [30]	60.42	67.95	78.93	61.60	75.93	7888	5124	193
JHIT [31]	79.21	82.29	86.91	89.80	85.37	2273	1058	177
NC2 [32]	71.85	74.81	87.30	78.52	85.84	2676	4554	159
EAFFMOT [22]	72.28	73.08	86.73	84.77	85.08	3946	1185	107
SpbTracker [33]	72.66	71.43	87.48	86.51	86.07	3508	875	257
UG3DMOT [34]	78.60	82.28	87.84	87.98	86.56	2993	1111	30
MMF-JDT [35]	79.52	84.01	87.65	88.06	86.24	2317	1751	37
CollabMOT [36]	80.02	81.86	87.14	91.70	85.77	2063	583	207
S3MOT [37]	76.86	77.41	87.87	86.93	86.60	1899	2053	543
C-TwiX [38]	77.58	78.84	86.95	89.68	85.50	2814	355	381
SG-LKF [39]	79.59	82.53	87.09	90.55	85.66	2741	348	160
Ours	80.13	84.20	88.55	87.93	87.45	3620	480	52

**Table 2:** Evaluation result of “Pedestrian” on KITTI test set.

Methods	HOTA↑	AssA↑	LocA↑	MOTA↑	MOTP↑	FP↓	FN↓	IDSW↓
Seg2Track-SAM2 [30]	44.40	50.51	78.93	37.81	69.31	8044	6058	296
StrongFusionMOT [7]	43.42	48.83	70.53	39.04	63.89	8727	5069	316
JHIT [31]	54.07	56.88	78.38	64.95	64.59	5903	1927	284
MSA-MOT [5]	44.73	49.34	71.21	47.86	64.35	7761	4101	209
C-TWiX [38]	52.44	54.35	79.15	64.95	75.28	6632	1160	381
RAM [40]	52.71	52.19	77.70	68.40	73.61	5394	1660	262
MO-YOLO [41]	51.46	58.39	77.86	56.84	73.74	8068	1759	164
EAFFMOT [22]	40.20	45.63	71.25	42.01	64.57	10,793	2431	201
APPTracker+ [42]	42.73	41.15	78.30	55.45	74.22	7936	2076	302
SpbTracker [33]	43.25	44.79	71.87	53.55	65.28	8591	1961	200
NC2 [32]	44.30	46.75	72.08	44.18	65.68	6176	6415	159
FNC2 [43]	46.55	46.68	72.07	56.05	65.68	6160	3679	333
MMTrack [44]	49.28	55.33	79.26	56.19	75.34	9081	886	175
Ours	53.24	58.53	78.49	62.34	74.47	7242	1336	141

**Table 3:** Ablation study on the proposed modules.

Methods	HOTA↑	MOTA↑	MOTP↑	FP↓	FN↓	IDSW↓	FPS↑
Baseline	77.449	87.275	86.602	1028	2722	83	110
Baseline + CIGTS	78.603	88.106	87.364	910	2642	69	117
Baseline + CIGTS + AMSLA	80.125	88.97	88.155	826	2317	50	128
Baseline + CIGTS + AMSLA + MFCTM	81.498	89.663	89.110	724	2228	36	135

**Table 4:** Ablation study on the confidence filtering threshold.

$\theta_{c}$	HOTA↑	MOTA↑	MOTP↑	FP↓	FN↓	IDSW↓
1	78.295	87.532	87.173	1047	2693	81
1.2	78.466	88.018	87.289	932	2668	72
1.4	78.603	88.106	87.364	910	2642	69
1.6	78.429	88.035	87.274	931	2669	72
1.8	78.237	87.486	87.036	1015	2716	85

**Table 5:** Ablation study on the confidence stratified threshold.

$\theta_{l}$	HOTA↑	MOTA↑	MOTP↑	FP↓	FN↓	IDSW↓
2.5	78.233	87.919	87.108	939	2677	76
3	78.425	87.998	87.215	924	2654	74
3.5	78.603	88.106	87.364	910	2642	69
4	78.435	88.002	87.234	931	2666	73
4.5	78.229	87.794	87.134	983	2694	77

**Table 6:** Ablation study on the activation threshold δ.

δ	HOTA↑	MOTA↑	MOTP↑	FP↓	FN↓	IDSW↓
1	78.207	87.736	87.160	937	2670	75
2	78.603	88.106	87.364	910	2642	69
3	78.383	87.973	87.191	931	2666	74

**Table 7:** Ablation study on the activation threshold ξ.

ξ	HOTA↑	MOTA↑	MOTP↑	FP↓	FN↓	IDSW↓
2	78.475	88.031	87.293	928	2673	72
3	78.603	88.106	87.364	910	2642	69
4	78.393	87.985	87.242	935	2687	74

**Table 8:** Ablation study on the association matrix.

$M_{g}$	$M_{r}$	$M_{\theta }$	HOTA↑	MOTA↑	MOTP↑	FP↓	FN↓	IDSW↓
√			79.589	88.496	87.800	884	2457	72
√	√		79.803	88.629	87.970	862	2403	66
√	√	√	80.125	88.970	88.155	826	2317	50

**Table 9:** Ablation study on matching space expansion factors.

μ	HOTA↑	MOTA↑	MOTP↑	FP↓	FN↓	IDSW↓
1.1	79.822	88.795	87.963	861	2355	57
1.3	79.994	88.887	88.076	845	2342	54
1.5	80.125	88.970	88.155	826	2317	50
1.7	80.031	88.912	88.097	837	2330	52
1.9	79.916	88.853	88.022	852	2342	55

**Table 10:** Ablation study on perception threshold.

μ	HOTA↑	MOTA↑	MOTP↑	FP↓	FN↓	IDSW↓
70	81.200	89.497	88.920	752	2268	44
75	81.375	89.560	89.045	736	2248	39
80	81.498	89.663	89.110	724	2228	36
85	81.405	89.605	89.052	735	2241	38
90	81.274	89.526	88.978	750	2259	42

## Data Availability

The data presented in this study are available in the multi-object tracking task of the KITTI project. These data were derived from the following resources available in the public domain: http://www.cvlibs.net/datasets/kitti/eval_tracking.php (accessed on 26 March 2025).
